# Correlation of common inflammatory cytokines with cognition impairment, anxiety, and depression in acute ischemic stroke patients

**DOI:** 10.1590/1414-431X2021e11517

**Published:** 2022-02-28

**Authors:** Rumei Li, Wenjun Fan, Dongmei Li, Xuesong Liu

**Affiliations:** 1 Department of Emergency, The Second Affiliated Hospital of Harbin Medical University, Harbin, China; 2 Department of Neurology, The Second Affiliated Hospital of Harbin Medical University, Harbin, China; 3 Nursing Department Office, The Second Affiliated Hospital of Harbin Medical University, Harbin, China

**Keywords:** Cognition impairment, Anxiety, Depression, Inflammatory cytokines, Acute ischemic stroke

## Abstract

Inflammatory cytokines are related to cognitive function and psychiatric disorders in patients with several diseases. However, few relevant studies have been performed on acute ischemic stroke (AIS) patients. Hence, this study aimed to investigate the correlation of common inflammatory cytokines with cognition impairment, anxiety, and depression in AIS patients. Common inflammatory cytokines of 176 AIS patients (including tumor necrosis factor-alpha (TNF-α), interleukin (IL)-1β, IL-6, and IL-17) were measured using Human Enzyme Linked Immunosorbent Assay Kits. Cognition impairment (Mini-Mental State Examination (MMSE)), anxiety (Hospital Anxiety and Depression Scale for anxiety (HADS-A)), and depression (HADS-D) were evaluated. The incidence of cognition impairment, anxiety, and depression was 43.2, 39.2, and 31.2%, respectively. TNF-α and IL-6 were negatively associated with MMSE score, and high TNF-α, IL-1β, and IL-6 were correlated with cognition impairment occurrence. In addition, TNF-α, IL-1β, and IL-17 were positively associated with HADS-A score, while only high TNF-α was associated with anxiety occurrence. Furthermore, TNF-α, IL-1β, and IL-17 were positively associated with HADS-D score, while high IL-1β, IL-6, and IL-17 correlated with depression occurrence. Multivariate logistic regression revealed that TNF-α and National Institutes of Health Stroke Scale (NIHSS) score ≥5 were associated with high risk of cognition impairment; TNF-α, IL-17, unemployed before surgery, hypertension, and chronic kidney disease (CKD) correlated with high anxiety occurrence. Furthermore, IL-17, divorced/widowed/single status, diabetes, and NIHSS score ≥5 were associated with high risk of depression. In conclusion, common inflammatory cytokines including TNF-α, IL-1β, and IL-17 were related to cognition impairment, anxiety, or depression in AIS patients.

## Introduction

Stroke is a huge health challenge characterized by the dysfunction and degeneration of brain vascular components, with over 2.5 million new cases in China annually (1). Acute ischemic stroke (AIS) is a major pathological type of stroke and occupies nearly 80% of new stroke cases (2,3). Although the recent decades have witnessed substantial advances in diagnostic and treatment options, AIS is still the leading cause of disability and one of the leading causes of mortality worldwide (4). Of note, several severe complications often occur to AIS patients, among which, cognitive impairment is a sort of neurological dysfunction manifestation presenting with dysmnesia, disorientation, decline of language competence, and so on, which directly decreases the quality of life of AIS patients (5,6). Other common psychological complications of AIS patients such as anxiety and depression have a negative influence on rehabilitation outcomes, neurotrophic agents' efficacy, and even mortality in AIS patients (7). Therefore, investigating potential indicators related to cognition impairment, anxiety, and depression is a prerequisite for developing effective therapies to improve outcomes of AIS patients.

Inflammation is considered a key step of the progression of ischemic stroke, which has been shown to increase brain injury, retard brain repair, and affect neurological outcomes (8,9). Interestingly, inflammation is also related to cognition impairment, anxiety, and depression in patients with several diseases. For instance, high serum creatinine-reactive protein is associated with depression occurrence in patients with isolated coronary artery ectasia (10). Also, increased TNF-α and IL-6 are correlated with high risk of mild cognitive impairment in type 2 diabetes patients (11). However, few relevant studies have been performed in AIS patients. Hence, this study aimed to investigate the correlation of common inflammatory cytokines with cognition impairment, anxiety, and depression in AIS patients.

## Material and Methods

### Patients

This study obtained approval from the Institutional Review Board of The Second Affiliated Hospital of Harbin Medical University. Between January 2019 and July 2020, 176 AIS patients who were admitted to this hospital were consecutively recruited for this study. Eligibility criteria for recruitment were as follows: i) diagnosis of AIS in line with the AIS guideline (12); ii) age more than 18 years; iii) volunteer to participate in the study and provide a blood sample for study use; and iv) able to complete the study assessment. Patients with any of the following conditions were considered ineligible for study enrollment: i) severe cognitive impairment, which was defined as Mini-Mental State Examination (MMSE) score <10; ii) presenting with intracranial hemorrhage; iii) known hematological diseases or active infections; iv) administered immunosuppressant within 3 months; v) complicated with inflammatory diseases; vi) had a history of malignancies; and vii) breast feeding or pregnant. All patients signed an informed consent prior to recruitment.

### Sample collection and analysis

Venous blood samples of patients were collected before they were discharged from the hospital after 12-h fasting, and the serum was separated within two hours. After blood collection, the tube was gently inverted and mixed 4-5 times, then put in the upright position at room temperature until the blood was completely coagulated (about 1 h). Following that, centrifugation was conducted at 1000 *g* for 10 min at room temperature, then serum was obtained. The collected serum was transferred to cryopreserved tubes and placed in a -70°C refrigerator. Levels of inflammatory cytokines in serum, including TNF-α, interleukin (IL)-1β, IL-6, and IL-17, were measured using Human Enzyme Linked Immunosorbent Assay (ELISA) kits (Invitrogen, USA). The ELISA was carried out following the manufacturer’s protocol.

### Data collection and evaluation

Clinical data collection was completed during hospitalization of patients, which mainly included sociodemographic information, smoking status, complications, as well as disease-related features. Assessment of cognition impairment, anxiety, and depression was conducted for all patients on the day of discharge. The cognition impairment status of patients was evaluated using MMSE, and a MMSE score ≤26 was considered as cognition impairment (13). The anxiety status and depression status of patients were assessed using the Hospital Anxiety and Depression Scale for anxiety (HADS-A) and the Hospital Anxiety and Depression Scale for depression (HADS-D), respectively. An HADS-A score >7 was indicative of anxiety, and similarly, a HADS-D score >7 was indicative of depression. Furthermore, the anxiety degree was classified as 8-10, mild anxiety; 11-14, moderate anxiety; and 15-21, severe anxiety (14). The depression degree was classified as 8-10, mild depression; 11-14, moderate depression; and 15-21, severe depression (14).

### Statistical analysis

SPSS 24.0 (IBM, USA) and GraphPad Prism 7.02 software (GraphPad Software Inc., USA) were applied for data analysis and diagram making. Descriptive analysis was performed for characteristics of patients, MMSE score, HADS-A score, and HADS-D score. Correlation analysis was determined by Spearman's rank correlation test. Comparison of differences was determined by chi-squared test or Wilcoxon rank sum test, as appropriate. All potential factors were included in the multivariate logistic regression analysis of cognition impairment, anxiety, and depression, and the independent factors were screened out by forward stepwise (conditional) method. A significant difference was indicated by a P value <0.05.

## Results

### AIS patients' characteristics

The detailed information about other characteristics is shown in Table 1. The mean age was 67.6±8.4 years, and there were 63.1% males and 36.9% females. With respect to marriage status, 47.2% patients were married and 52.8% patients were divorced/widowed/single. As for employment status before AIS, 11.4% patients were employed and 88.6% patients were unemployed. In addition, 27.3% patients were current smokers, 84.7% patients had hypertension, 50.0% patients had hyperlipidemia, 36.4% patients had diabetes mellitus, and 13.6% patients had CKD. Furthermore, 40.3, 34.1, and 25.6% of patients had left, right, and bilateral brainstem unknown lesion location, respectively. The mean NIHSS score was 7.0±3.0.

**Table 1:** Characteristics of acute ischemic stroke (AIS) patients.

Items	AIS patients (n=176)
Age (years), mean±SD	67.6±8.4
Gender, n (%)	
Male	111 (63.1)
Female	65 (36.9)
Education duration (years), median (IQR)	7.0 (5.3-9.0)
Marriage status, n (%)	
Married	83 (47.2)
Divorced/widowed/single	93 (52.8)
Employment status before AIS, n (%)	
Employed	20 (11.4)
Unemployed	156 (88.6)
Current smoking, n (%)	
No	128 (72.7)
Yes	48 (27.3)
Hypertension, n (%)	
No	27 (15.3)
Yes	149 (84.7)
Hyperlipidemia, n (%)	
No	88 (50.0)
Yes	88 (50.0)
Diabetes mellitus, n (%)	
No	112 (63.6)
Yes	64 (36.4)
CKD, n (%)	
No	152 (86.4)
Yes	24 (13.6)
Lesion location, n (%)	
Left	71 (40.3)
Right	60 (34.1)
Bilateral/brainstem/unknown	45 (25.6)
NIHSS score*, mean±SD	7.0±3.0

SD: standard deviation; IQR: interquartile range; CKD: chronic kidney disease; NIHSS: National Institutes of Health Stroke Scale. *NIHSS score was evaluated within 24 h after admission.

### Cognition impairment, anxiety, and depression assessment

**Figure 1:**
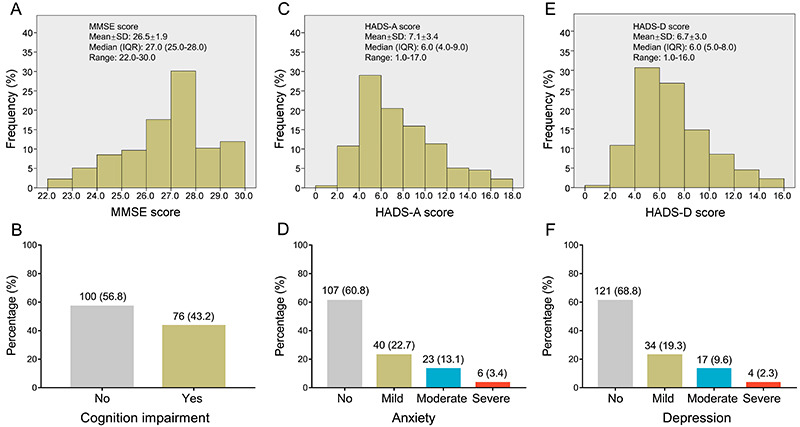
Overall cognition impairment, anxiety, and depression in acute ischemic stroke patients. Distribution of MMSE score (**A**), HADS-A score (**C**), and HADS-D score (**E**). Number (percentage) of cognition impairment (**B**), anxiety (**D**), and depression (**F**) grade. MMSE: Mini-Mental State Examination; HADS-A: Hospital Anxiety and Depression Scale for anxiety; HADS-D: Hospital Anxiety and Depression Scale for depression; SD: standard deviation; IQR: interquartile range.

In regard to cognition impairment, the mean value, median value, and the range of MMSE were 26.5±1.9, 27.0 (IQR: 25.0-28.0), and 22.0-30.0, respectively (Figure 1A). There were 43.2% patients with cognition impairment, and 56.8% patients with no cognition impairment (Figure 1B). Regarding anxiety, the mean value, median value, and the range of HADS-A were 7.1±3.4, 6.0 (IQR: 4.0-9.0), and 1.0-17.0, respectively (Figure 1C). There were 60.8, 22.7, 13.1, and 3.4% of patients with a grade of no anxiety, mild anxiety, moderate anxiety, and severe anxiety (Figure 1D). In addition, the mean value, median value, and the range of HADS-D were 6.7±3.0, 6.0 (IQR: 5.0-8.0), and 1.0-16.0, respectively (Figure 1E); There were 68.8, 19.3, 9.6, and 2.3% of patients with no depression, mild depression, moderate depression, and severe depression, respectively (Figure 1F).

### Characteristics associated with cognition impairment, anxiety, and depression

Cognition impairment occurrence was correlated with older age (P=0.035), hyperlipidemia (P=0.033), and NIHSS score ≥5 (P<0.001). Furthermore, anxiety occurrence was associated with divorced/widowed/single status (P=0.043), unemployed status (P=0.019), hypertension (P=0.005), diabetes mellitus (P<0.001), and CKD (P=0.012). In addition, depression occurrence was related to divorced/widowed/single status (P=0.010), diabetes mellitus (P=0.001), CKD (P=0.009), and high NIHSS score (P=0.008) (Table 2).

**Table 2:** Cognition impairment, anxiety, and depression rate among acute ischemic stroke (AIS) patients with different clinical features.

Clinical features	Cognition impairment	Anxiety	Depression
Age, n (%)			
<70 years	35 (36.1)	34 (35.1)	28 (28.9)
≥70 years	41 (51.9)	35 (44.3)	27 (34.2)
P value	**0.035**	0.211	0.450
Gender, n (%)			
Male	53 (47.7)	42 (37.8)	36 (32.4)
Female	23 (35.4)	27 (41.5)	19 (29.2)
P value	0.110	0.627	0.658
Education duration, n (%)			
<7 years	35 (43.2)	28 (34.6)	21 (25.9)
≥7 years	41 (43.2)	41 (43.2)	34 (35.8)
P value	0.994	0.245	0.159
Marriage status, n (%)			
Married	34 (41.0)	26 (31.3)	18 (21.7)
Divorced/widowed/single	42 (45.2)	43 (46.2)	37 (39.8)
P value	0.159	**0.043**	**0.010**
Employment status before AIS, n (%)			
Employed	7 (35.0)	3 (15.0)	5 (25.0)
Unemployed	69 (44.2)	66 (42.3)	50 (32.1)
P value	0.433	**0.019**	0.522
Current smoking, n (%)			
No	60 (46.9)	54 (42.2)	42 (32.8)
Yes	16 (33.3)	15 (31.3)	13 (27.1)
P value	0.106	0.186	0.465
Hypertension, n (%)			
No	8 (29.6)	4 (14.8)	5 (18.5)
Yes	68 (45.6)	65 (43.6)	50 (33.6)
P value	0.122	**0.005**	0.121
Hyperlipidemia, n (%)			
No	31 (35.2)	35 (39.8)	26 (29.5)
Yes	45 (51.1)	34 (38.6)	29 (33.0)
P value	**0.033**	0.877	0.626
Diabetes mellitus, n (%)			
No	45 (40.2)	33 (29.5)	25 (22.3)
Yes	31 (48.4)	36 (56.3)	30 (46.9)
P value	0.287	**<0.001**	**0.001**
CKD, n (%)			
No	68 (44.7)	54 (35.5)	42 (27.6)
Yes	8 (33.3)	15 (62.5)	13 (54.2)
P value	0.295	**0.012**	**0.009**
Lesion location, n (%)			
Left	30 (42.3)	31 (43.7)	23 (32.4)
Right	28 (46.7)	21 (35.0)	19 (31.7)
Bilateral/brainstem/unknown	18 (40.0)	17 (37.8)	13 (28.9)
P value	0.776	0.584	0.921
NIHSS score*, n (%)			
<5	14 (23.3)	18 (30.0)	11 (18.3)
≥5	62 (53.4)	51 (44.0)	44 (37.9)
P value	**<0.001**	0.072	**0.008**

CKD: chronic kidney disease; NIHSS: National Institutes of Health Stroke Scale. *NIHSS score was evaluated within 24 h after admission. Bold type indicates statistical significance (chi-squared test or Wilcoxon rank sum test).

### Correlation of inflammatory cytokines with cognition impairment

**Figure 2:**
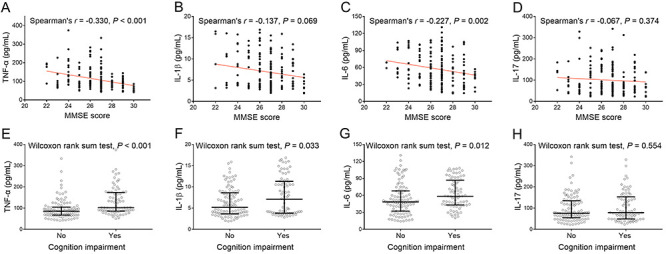
Common inflammatory cytokines were positively associated with cognition impairment in acute ischemic stroke patients. Correlation of TNF-α, IL-1β, IL-6, and IL-17 with MMSE score (**A**-**D**) and with cognition impairment occurrence (**E**-**H**). Lines indicate median and IQR. TNF-α: tumor necrosis factor alpha; IL: interleukin; MMSE: Mini-Mental State Examination.

TNF-α (P<0.001) and IL-6 (P=0.002), but not IL-1β (P=0.069) or IL-17 (P=0.374), were negatively associated with MMSE score. In addition, high TNF-α (P<0.001, adjusted P <0.001), IL-1β (P=0.033, adjusted P=0.132), and IL-6 (P=0.012, adjusted P=0.048), but not IL-17 (P=0.554, P=1.000 after adjustment) were correlated with cognition impairment occurrence (Figure 2A-H).

### Correlation of inflammatory cytokines with anxiety

**Figure 3:**
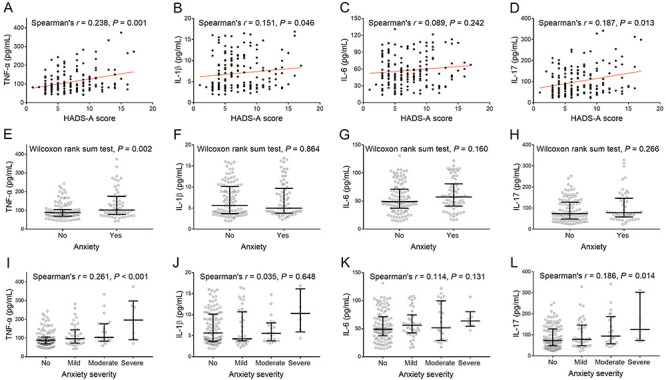
Common inflammatory cytokines were positively associated with anxiety in acute ischemic stroke patients. Correlation of TNF-α, IL-1β, IL-6, and IL-17 with HADS-A (**A**-**D**), with anxiety occurrence (**E**-**H**), and with anxiety severity (**I**-**L**). Lines indicate median and IQR. TNF-α: tumor necrosis factor alpha; IL: interleukin; HADS-A: Hospital Anxiety and Depression Scale for anxiety.

TNF-α (P=0.001), IL-1β (P=0.046), and IL-17 (P=0.013), but not IL-6 (P=0.242), were positively associated with HADS-A score. High TNF-α (P=0.002, adjusted P=0.008), but not high IL-1β (P=0.864, adjusted P=1.000), IL-6 (P=0.160, adjusted P=0.640), and IL-17 (P=0.266, adjusted P=1.000), was correlated with anxiety occurrence. Furthermore, high TNF-α (P<0.001, adjusted P <0.001) and increased IL-17 (P=0.014, adjusted P=0.056), but not IL-1β (P=0.648, adjusted P=1.000) or IL-6 (P=0.131, adjusted P=0.524), were correlated with increased anxiety severity grade (Figure 3A-L).

### Correlation of inflammatory cytokines with depression

**Figure 4:**
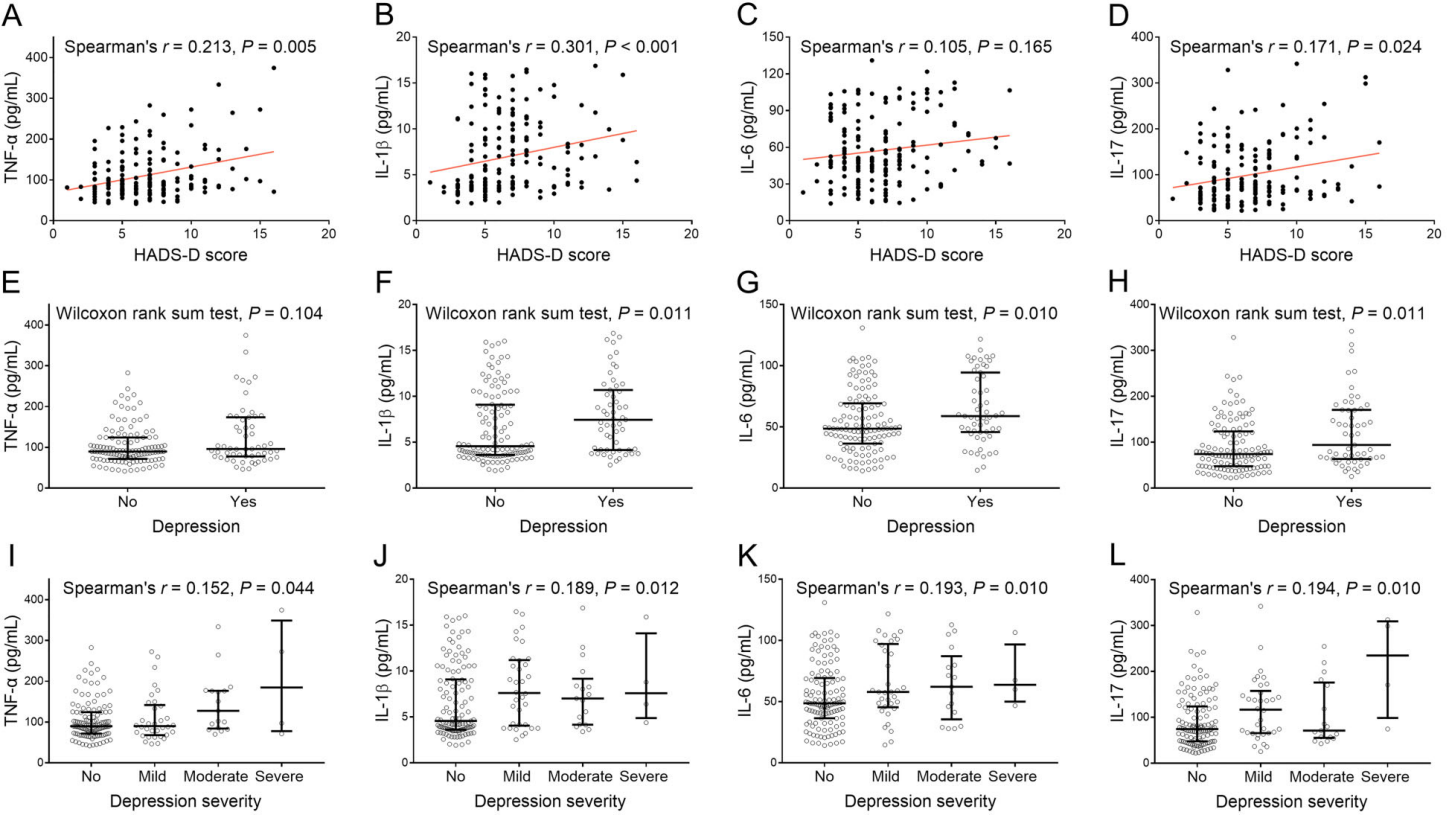
Common inflammatory cytokines were positively associated with depression in acute ischemic stroke patients. Correlation of TNF-α, IL-1β, IL-6, and IL-17 with HADS-A (**A**-**D**), with depression occurrence (**E**-**H**), and with depression severity (**I**-**L**). Lines indicate median and IQR. TNF-α: tumor necrosis factor alpha; IL: interleukin; HADS-D: Hospital Anxiety and Depression Scale for depression.

TNF-α (P=0.005), IL-1β (P<0.001), and IL-17 (P=0.024), but not IL-6 (P=0.165), were positively associated with HADS-D score. High IL-1β (P=0.011, adjusted P=0.044), IL-6 (P=0.010, adjusted P=0.040), and IL-17 (P=0.011, P=0.044 after adjustment), but not TNF-α (P=0.104, P=0.416 after adjustment), were associated with depression occurrence. Furthermore, high TNF-α (P=0.044, P=0.176 after adjustment), high IL-1β (P=0.012, P=0.048 after adjustment), increased IL-6 (P=0.010, P=0.040 after adjustment), and increased IL-17 (P=0.010, P=0.040 after adjustment) were correlated with increased depression severity grade (Figure 4A-L).

### Independent risk factors related to cognition impairment, anxiety, and depression

Multivariate logistic regression showed that TNF-α (OR=2.882, P=0.004) and NIHSS score ≥5 (OR=1.009, P=0.004) were associated with high risk of cognition impairment. TNF-α (OR=1.007, P=0.025), IL-17 (OR=1.008, P=0.007), unemployed before surgery (OR=4.099, P=0.046), hypertension (OR=5.283, P=0.007), and CKD (OR=3.224, P=0.026) were associated with high risk of anxiety. Furthermore, IL-17 (OR=1.007, P=0.009), divorced/widowed/single status (OR=2.668, P=0.008), diabetes (OR=3.337, P=0.001), and NIHSS score ≥5 (OR=2.370, P=0.036) were associated with high risk of depression (Table 3).

**Table 3:** Independent factors associated with cognition impairment, anxiety, and depression of acute ischemic stroke (AIS) patients.

Parameters	Multivariate logistic regression^#^					
	SE	Wald	P value	OR	95% CI	
					Lower	Upper
Cognition impairment						
TNF-α	0.371	8.161	0.004	2.882	1.394	5.958
NIHSS* score ≥5 (<5 as ref.)	0.003	8.080	0.004	1.009	1.003	1.016
Anxiety						
TNF-α	0.003	5.042	0.025	1.007	1.001	1.014
IL-17	0.003	7.168	0.007	1.008	1.002	1.014
Unemployed before surgery (Employed as ref.)	0.706	3.997	0.046	4.099	1.028	16.345
Hypertension (No hypertension as ref.)	0.621	7.173	0.007	5.283	1.563	17.861
CKD (no CKD as ref.)	0.527	4.927	0.026	3.224	1.147	9.065
Depression						
IL-17	0.003	6.867	0.009	1.007	1.002	1.013
Divorced/widowed/single (married as ref.)	0.372	6.941	0.008	2.668	1.286	5.537
Diabetes (no diabetes as ref.)	0.366	10.869	0.001	3.337	1.630	6.832
NIHSS score* ≥5 (<5 as ref.)	0.412	4.390	0.036	2.370	1.057	5.314

SE: standard error; OR: odds ratio; CI: confidence interval; TNF-α: tumor necrosis factor alpha; NIHSS: National Institutes of Health Stroke Scale; IL-17: interleukin 17; CKD: chronic kidney disease. *NIHSS score was evaluated within 24 h after admission. ^#^All potential factors were included in the multivariate logistic regression analysis, and the independent factors were screened out by forward stepwise (conditional) method.

## Discussion

In the current study, the incidence of cognition impairment, anxiety, and depression was 43.2, 39.2, and 31.2% in AIS patients. After acute ischemic insult, inflammatory cytokines in the ischemic brain will be upregulated from resident brain cells and infiltrating immune cells, which play complex roles in the pathophysiology of cerebral ischemia (9). Previous evidence supports a relationship of cognition impairment, anxiety, and depression with inflammation processes in several diseases (such as coronary heart patients and cancer) (10,15,16). However, few studies have been performed on the potential cognition impairment/anxiety/depression-associated inflammatory cytokines in AIS patients. Hence, deeply understanding the clinical implication of inflammatory cytokines on cognition impairment/anxiety/depression is a precondition to improve the prognosis of AIS patients. In the present study, we discovered that high TNF-α and IL-6 were correlated with cognition impairment occurrence; high TNF-α, IL-1β, and IL-17 were correlated with anxiety or depression occurrence in AIS patients. The possible reasons were that: 1) TNF-α and IL-6, as pro-inflammatory cytokines, could cross the blood-brain barrier by a transport system, thereby promoting communication between the central nervous system and the periphery (17,18). Hence, high concentrations of TNF-α and IL-6 might be important factors for promoting the development of cognition impairment, thereby increased cognition impairment occurrence; and 2) a high level of common pro-inflammatory cytokines (including TNF-α, IL-1β, and IL-17) might affect indoleamine 2,3-deoxygenation enzyme-1 (IDO1), subsequently influence 5-hydroxytryptamine (5-HT) (an important neurotransmitter related to depression), and eventually increase anxiety and depression occurrence in AIS patients (19-22).

In addition, TNF-α and NIHSS score ≥5 were related to a high risk of cognition impairment. Also, IL-17, unemployed before surgery, hypertension, CKD, divorced/widowed/single status, diabetes, and NIHSS score ≥5 were correlated with a high risk of anxiety or depression in AIS patients. The probable explanations were as follows: 1) Regarding cognition impairment, TNF-α served as a common inflammation cytokine, and the possible reason for its correlation with cognition impairment was as mentioned above; high NIHSS score meant worse neurological status in AIS patients, and cognition impairment was also a kind of manifestation of neurological dysfunction, hence, an NIHSS score ≥5 was an independent risk factor for cognition impairment. 2) As for anxiety and depression, the impact of IL-17 on anxiety and depression was also as mentioned above. Furthermore, unemployed before surgery and divorced/widowed/single status meant unfavorable social status, which might make patients feel less capable and alone, increasing stress after AIS, thereby causing a high risk of anxiety and depression. Meanwhile, patients with severe complications (including hypertension, CKD, and diabetes) are under long-term metabolic dysfunction, which might affect their immune system and increase their negative stress, thereby leading to a high risk of anxiety and depression in AIS patients. In addition, an NIHSS score ≥5 meant worse neurological status in AIS patients, which also produces huge stress, and eventually, increased anxiety and depression occurrence in AIS patients.

Several limitations existed in this study. Although the potential cognition impairment/anxiety/depression-associated inflammatory cytokines in AIS patients have been explored, the detailed mechanisms of these inflammatory cytokines underlying cognition impairment/anxiety/depression of AIS patients still remain unclear. Further relevant study is needed.

In conclusion, common inflammatory cytokines including TNF-α, IL-1β, and IL-17 were related to cognition impairment, anxiety, and depression in AIS patients in this study.
